# Learning Curve Analysis of Alpha DaRT Implantation for Cutaneous Malignancies: A Single-Surgeon Experience

**DOI:** 10.3390/cancers18213425

**Published:** 2026-10-22

**Authors:** Tomer Charas, Yotam Shkedy

**Affiliations:** 1 Radiation Oncology Department, Rambam Health Care Campus, Haifa 3109601, Israel; 2 ORL and Audiology Department, Counties Manukau, Te Whatu Ora/Health New Zealand, Auckland 2104, New Zealand; yotam.shkedy@middlemore.co.nz

**Keywords:** Diffusing Alpha-emitters Radiation Therapy (Alpha DaRT), alpha-particle therapy, basal cell carcinoma, squamous cell carcinoma, cutaneous malignancies, learning curve, procedural efficiency, interstitial radiotherapy, radiation oncology

## Abstract

**Background/Objectives**: Diffusing Alpha-emitters Radiation Therapy (Alpha DaRT) is a novel interstitial radiotherapy technique that delivers high-linear energy transfer alpha radiation directly into tumor tissue. As with any new procedural oncology intervention, operator experience may influence procedural efficiency and reproducibility. This study evaluated the learning curve associated with Alpha DaRT implantation for cutaneous malignancies using procedure duration as a surrogate measure of procedural efficiency. **Methods**: A retrospective learning-curve analysis was conducted using procedural data from a prospective, single-arm clinical trial (NCT03889899). Alpha DaRT implantation procedures performed by a single surgeon in 24 patients with basal-cell carcinoma and squamous-cell carcinoma were analyzed. Given the variability in procedure duration due to lesion size, duration per applicator was used as a standardized endpoint for learning-curve assessment. Surgeon experience was defined by sequential procedure number, and sequential regression analyses evaluated changes over time. **Results**: Twenty-six Alpha DaRT procedures were performed between October 2020 and February 2025. A moderate negative correlation was observed between procedure number and duration (Spearman’s r = −0.57, *p* = 0.002). The greatest reduction in duration was observed after the first five procedures, with mean duration per applicator decreasing from 221 s in the initial cases to 153 s thereafter. Tumor volume was independently associated with longer procedural duration (*p* < 0.001), while lesion type, location, and number of applicators were not. **Conclusions**: Alpha DaRT implantation demonstrated a short initial learning curve when assessed by procedural efficiency, with substantial improvement observed after approximately five procedures. These findings suggest that procedural familiarity develops rapidly; however, validation across multiple operators and institutions is needed to determine the generalizability of these results and inform future training standards.

## Simple Summary

Diffusing alpha-emitter radiation therapy is a new cancer treatment that delivers highly targeted radiation directly to tumors while minimizing damage to nearby healthy tissue. As this treatment becomes more widely used, it is important to understand how quickly clinicians become proficient in performing the implantation procedure. This study examined the relationship between physician experience and normalized procedure duration during the early clinical use of this therapy for skin and other superficial tumors. Procedure times decreased as operator experience increased, with the greatest improvement occurring during the first few procedures. These findings suggest that procedural efficiency can be achieved relatively quickly and provide important information for training, clinical implementation, and future studies. Understanding the learning curve associated with this treatment may support its safe and effective adoption in routine cancer care.

## 1. Introduction

Diffusing Alpha-emitters Radiation Therapy (Alpha DaRT) is an emerging therapeutic modality designed to deliver highly localized alpha-particle radiation directly into tumor tissue. The system utilizes radium-224-embedded sources that release short-lived alpha-emitting daughter isotopes capable of limited diffusion within the tumor microenvironment. The resulting high-linear energy transfer (LET) radiation enables highly localized energy deposition within tumor tissue while minimizing exposure to surrounding normal tissues because of the very short penetration depth of alpha particles. At the cellular level, this localized irradiation induces complex, often irreparable double-stranded DNA damage that contributes to tumor cell death. Beyond the extent of DNA damage, the biological effectiveness of alpha radiation is also influenced by the manner in which that damage is produced. In contrast to conventional low-LET radiation modalities, alpha particles generate dense ionization tracks that result in a greater proportion of direct DNA strand breaks rather than indirect damage mediated by reactive oxygen species. Owing to the high density of energy deposition along their path, alpha particles frequently induce multiple DNA lesions within a confined region of the cell nucleus, including complex double-strand breaks and clustered sites of damage. These highly localized and concentrated DNA insults present a substantial challenge to cellular repair mechanisms and are therefore associated with increased biological effectiveness compared with sparsely ionizing forms of radiation. As a result, alpha-particle irradiation may maintain therapeutic effectiveness even in tumor cells that exhibit relative resistance to conventional low-LET radiation treatments [1]. Furthermore, the diffusion of short-lived daughter isotopes from the implanted sources enables irradiation of tumor cells beyond the immediate vicinity of the source, potentially improving dose distribution within heterogeneous tumor tissues. This unique combination of highly localized energy deposition and potent radiobiological activity allows therapeutic radiation doses to be concentrated within the tumor while limiting exposure to adjacent normal tissues [2]. Collectively, these radiobiological and dosimetric properties contribute to the potent local antitumor effects of Alpha DaRT while maintaining a favorable profile of radiation exposure to adjacent healthy tissues [2].

Unlike conventional brachytherapy, in which radiation is delivered primarily from fixed source positions, Alpha DaRT relies on the controlled diffusion of short-lived alpha-emitting daughter isotopes from implanted Ra-224 sources. This mechanism enables irradiation of tumor tissue beyond the immediate source track and facilitates coverage of irregularly shaped lesions while maintaining the highly localized dose deposition characteristic of alpha particles. Consequently, treatment planning requires careful consideration of source distribution, spacing, and tumor geometry to achieve adequate target coverage and dose distribution [3].

Alpha DaRT treatment involves the implantation of radium-224-loaded sources directly within the tumor according to a patient-specific treatment plan designed to achieve the prescribed radiation dose distribution. Successful implementation requires accurate source positioning, appropriate spacing and orientation of applicators, and adherence to predefined dosimetric parameters to ensure adequate tumor coverage while minimizing radiation exposure to surrounding healthy tissues. Depending on lesion size, shape, depth, and anatomical location, the treating physician may need to determine the optimal number of implantation tracks, needle trajectories, and source locations required to achieve the intended treatment geometry. Needle insertion is technically challenging in anatomically constrained areas or when treating irregularly shaped lesions, requiring careful consideration of source spacing and placement to maintain adequate target coverage. Deviations from the planned implantation geometry may affect dose distribution and treatment consistency, emphasizing the importance of procedural precision throughout the implantation process. The procedure therefore combines elements of treatment planning, spatial judgment, and technical implantation skills, making it dependent on consistent execution by the treating physician. As with other image-guided and interventional oncologic procedures, increasing operator experience may influence procedural efficiency, workflow familiarity, and technical performance, highlighting the importance of characterizing the learning curve during clinical adoption of this novel therapeutic modality [4].

Clinical studies have demonstrated encouraging safety and efficacy outcomes for Alpha DaRT in the management of non-melanoma skin cancers, particularly basal-cell carcinoma (BCC) and squamous-cell carcinoma (SCC), including lesions that are recurrent, unresectable, or poorly suited for conventional surgery or external beam radiotherapy [5,6,7]. Reported findings have demonstrated favorable local tumor control, together with an acceptable safety profile, supporting further clinical development of the technology across a range of oncologic applications [8,9]. As Alpha DaRT moves toward broader clinical adoption, understanding the procedural learning curve associated with its implantation is essential.

The learning curve is a critical determinant in the safe implementation of novel interventional oncology techniques [10,11]. Prior literature across surgical, interventional, and radiation oncology disciplines has consistently demonstrated associations between operator experience, procedural efficiency, and clinical outcomes [12,13]. Procedural duration can frequently be used as a surrogate measure of efficiency and technical familiarity, particularly in early assessments of novel procedures [14]. Although procedural performance may also encompass measures of accuracy and clinical outcomes, procedural duration provides a practical and objective metric for evaluating early operator adaptation to a new technique [15,16]. Learning-curve analyses across a range of procedural disciplines have frequently utilized procedure time and efficiency-related measures as quantifiable indicators of skill acquisition and technical familiarity [17,18]. More broadly, procedural accuracy, efficiency, and treatment outcomes provide objective measures of operator performance, and for innovative therapies such as Alpha DaRT, systematic assessment of these factors is essential for successful clinical adoption, standardized implementation, and consistent outcomes in multicenter studies.

Evidence from different procedures across multiple disciplines demonstrates this principle, showing that operator experience and repeated practice directly influence efficiency, accuracy, and outcome consistency. Use of modular endoscopes for sialendoscopy was associated with reductions in procedure duration, with average operative times decreasing from 39 min during the first 10 cases to shorter times over the next 20 cases, and continued improvement observed in subsequent procedures [19]. Similarly, a robot-assisted spine surgery demonstrated a clear learning curve, with average total operative time decreasing from 165 min during the first 30 cases to 112 min in subsequent cases, reaching a plateau around case 14 [20]. Comparable learning-curve effects have been described in HDR prostate brachytherapy, a procedure that similarly requires accurate source placement and treatment planning. Increasing operator experience was associated with improvements in procedural workflow and implementation efficiency, supporting the concept that proficiency in radiation-based implantation procedures develops progressively with repeated clinical experience [21]. Similar findings have been reported for CT-guided radioactive seed implantation, where procedural efficiency improved with operator experience and the learning curve reached a plateau after approximately 20 cases, highlighting the importance of experience in image-guided radiation implantation techniques [22]. Collectively, these observations highlight the importance of procedure-specific learning curves and support the need to characterize the acquisition of technical proficiency during the clinical implementation of novel therapies such as Alpha DaRT.

Despite growing clinical interest in Alpha DaRT, data describing operator learning curves and procedural efficiency remain limited. This analysis aimed to quantify the relationship between procedural experience and implantation efficiency during early clinical use of Alpha DaRT for cutaneous malignancies. Exploratory analyses evaluated changes in procedure duration and implantation efficiency across sequential implantation procedures as surrogate measures of increasing operator familiarity with the technique.

## 2. Materials and Methods

This retrospective learning-curve analysis was conducted using procedural data from an open-label, single-arm prospective clinical trial (NCT03889899) evaluating the safety and efficacy of Alpha DaRT for malignant cutaneous, mucosal, and superficial soft-tissue neoplasms. All procedures included in this analysis were performed at Rambam Medical Center (Haifa, Israel) by a single experienced otolaryngology surgeon.

Alpha DaRT was delivered using Alpha Flex applicators (Jerusalem, Israel), consisting of preloaded flexible tubes containing between one and five radium-224 sources per applicator. Applicators were inserted interstitially into the target lesion under standard institutional protocols following lesion mapping and treatment planning. Applicators could be curved or straight depending on anatomical constraints and tumor geometry.

Clinical records were reviewed to extract procedural and tumor-related variables. Procedural efficiency was assessed using procedure duration normalized per applicator (seconds/applicator), calculated by dividing the total implantation time by the number of applicators implanted during the procedure. Procedure duration reflected the implantation phase and did not include pre-procedural planning or post-implant care.

Surgeon experience was indexed by sequential procedure number, beginning with the first Alpha DaRT procedure performed at the institution. Lesion characteristics collected included histologic subtype (BCC or SCC), anatomical location, longest lesion diameter, gross tumor volume, lesion status (newly diagnosed vs. recurrent/persistent), number of applicators used, and total number of sources implanted.

All statistical analyses were conducted using SAS software (Version 9.4, SAS Institute, Cary, NC, USA). Spearman’s correlation coefficients were computed to explore relationships between procedure duration and potential explanatory variables. Linear regression models were used to evaluate covariate effects on procedure duration. To identify a potential learning-curve threshold, sequential procedure-number cutoffs were evaluated in separate regression models adjusted for diagnosis (BCC vs. SCC), disease status (newly diagnosed, persistent, or recurrent), number of sources, number of applicators, longest lesion diameter, and gross tumor volume (GTV). The cutoff associated with the lowest *p*-value was selected as the threshold providing the greatest differentiation in procedure duration between groups. Because this analysis was exploratory and intended to identify the point of maximal separation between candidate thresholds, no adjustment for multiple testing was performed.

To explore the procedural learning curve, sequential regression analyses were performed using dichotomous indicators representing procedures performed before versus after each potential cutoff number. Candidate cutoff values ranging from 2 to 9 procedures were evaluated separately. In each model, procedure duration served as the dependent variable, while the cutoff indicator was included as the primary independent variable and adjusted for diagnosis (BCC vs. SCC), disease status (newly diagnosed, persistent, or recurrent), number of sources, number of applicators, longest lesion diameter, and gross tumor volume (GTV). The cutoff producing the largest before-and-after difference in procedure duration was identified as the point at which the greatest efficiency gain occurred. Statistical significance was defined as *p* < 0.05.

## 3. Results

### 3.1. Patient and Lesion Characteristics

Between October 2020 and February 2025, 26 Alpha DaRT implantation procedures were performed in 24 patients. Median patient age was 71 years (range, 37–94 years), with 15 males (62.5%) and 9 females (37.5%), as shown in Table 1. Two patients underwent treatment for two distinct lesions during separate implantation procedures. Due to the procedure analysis being evaluated at the procedure level, each implantation was considered an independent observation.

Among the 26 lesions, 17 (65%) were BCC and 9 (35%) were SCC. Ten lesions (38%) were newly diagnosed, while the remainder were recurrent or persistent following prior treatment. Lesion locations varied and included the nose, ear, scalp, lip, forehead, cheek, and lower extremities. The longest lesion diameters ranged from 1.0 to 4.17 cm, and gross tumor volumes ranged from 0.06 to 7.31 cm^3^, as shown in Table 2.

### 3.2. Learning Curve and Procedural Efficiency

Procedural efficiency was evaluated using implantation duration normalized per applicator (seconds/applicator) to account for differences in the number of applicators used across procedures. Although total procedure duration varied greatly depending on tumor size, tumor location and other tumor-specific parameters, all learning-curve analyses were based on normalized implantation duration.

Normalized procedure duration demonstrated a moderate negative correlation with procedure number (Spearman’s r = −0.57, *p* = 0.002), indicating decreasing duration with increasing operator experience. No statistically significant correlations were identified between procedure duration and lesion type, anatomical location, recurrence status, number of applicators, or number of sources implanted, as shown in Table 3 (for raw data, see Table S1).

**Figure 1:**
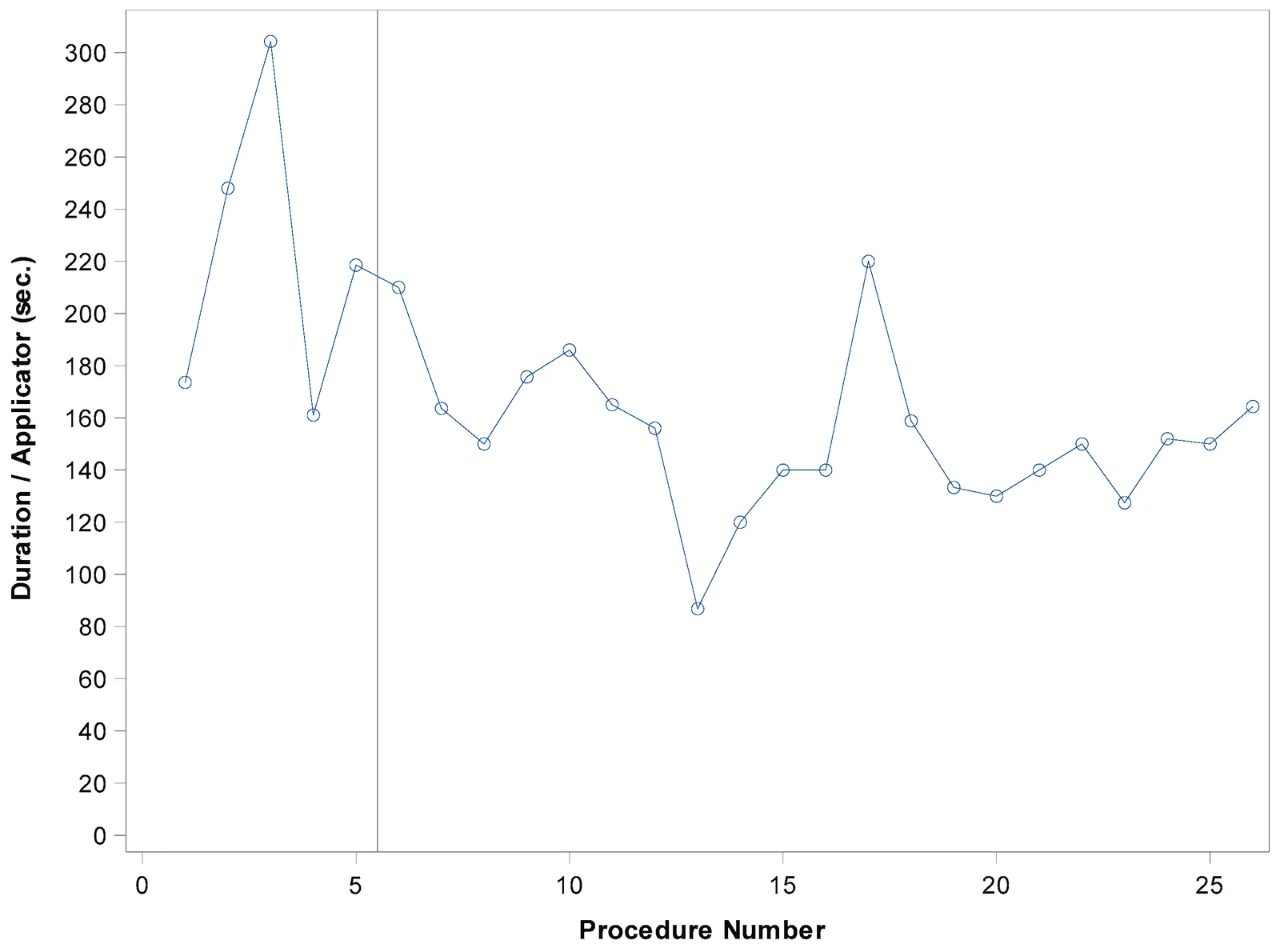
Duration per applicator (in seconds) with increased surgeon experience (subsequent procedure number) showing higher durations in the first 5 procedures than procedures thereafter. The vertical reference line separates between the first 5 procedures and the latter procedures.

Sequential regression analysis revealed that the most pronounced reduction in normalized procedure duration occurred after the fifth procedure, with a highly significant effect of the dichotomous cutoff variable (*p* < 0.0001). The first five procedures had a mean duration of 221 s per applicator, compared with 153 s per applicator for all subsequent procedures. Furthermore, variation in procedure duration decreased notably from the sixth procedure onward as evidenced by the drop in standard deviation. Although duration continued to trend downward beyond the cutoff point, the steepest efficiency gain was observed early in the experience, as shown in Figure 1.

A multivariable analysis incorporating procedural, clinical, and tumor-related characteristics was performed to identify factors associated with procedure duration. Of all the variables evaluated, gross tumor volume was the only independent predictor of longer procedure duration (*p* < 0.001).

## 4. Discussion

This study provides an early characterization of the learning curve associated with Alpha DaRT implantation for cutaneous malignancies. Using normalized procedure duration as a surrogate for procedural efficiency, a clear reduction in implantation time was observed with increasing experience, with the most substantial improvement occurring during the first five cases.

Learning curves of comparable length have been reported for other technically complex procedures with substantial variability across advanced surgical technologies [23,24]. Collectively, these findings suggest that familiarity with procedural workflow, instrument handling, and spatial planning, rather than tumor-specific complexity alone, may play a central role in early efficiency gains.

Importantly, procedural duration reflects only one dimension of technical proficiency. While improved efficiency may translate into reduced patient discomfort and optimized resource utilization, it should not be equated directly with procedural quality or oncologic effectiveness. Furthermore, learning curves in oncologic procedures are not always characterized by progressively shorter procedure times. As operator experience increases, clinicians may undertake more technically complex cases, which can influence procedure duration independently of proficiency. Similar findings have been reported in retroperitoneal sarcoma surgery, where operative times did not continue to decrease throughout the learning curve and were influenced by increasing case complexity [25]. The present analysis did not assess implantation accuracy, dosimetric parameters, or oncologic outcomes as learning-curve endpoints.

Although procedure duration provides an objective and readily quantifiable measure of procedural efficiency, operator proficiency in radiation-based implantation procedures is inherently multidimensional. Additional measures, including implantation quality, target coverage, dosimetric consistency, toxicity outcomes, and local tumor control, may provide complementary insights into procedural performance [26]. Evaluation of these parameters may help distinguish improvements attributable primarily to workflow familiarity from those reflecting meaningful gains in technical proficiency. For example, operators may become more efficient in treatment execution while simultaneously refining source placement accuracy, improving adherence to planned implantation geometry, and reducing procedural variability. Assessment of these endpoints could therefore provide a more comprehensive understanding of how procedural experience translates into treatment quality and clinical effectiveness. As clinical experience with Alpha DaRT expands and larger multicenter datasets become available, future learning-curve analyses incorporating these parameters may help further characterize the relationship between procedural efficiency, technical quality, and clinical outcomes. Such investigations may provide a more comprehensive understanding of the relationship between procedural experience, treatment quality, and clinical outcomes in radiation-based implantation procedures [27]. Such assessments could contribute to the development of standardized training frameworks and competency benchmarks for Alpha DaRT implantation.

Previous clinical investigations have provided encouraging evidence supporting the safety, feasibility, and therapeutic effectiveness of Alpha DaRT, with reported toxicities generally limited to mild-to-moderate local reactions and no significant late treatment-related toxicities observed during long-term follow-up. In parallel with the growing clinical experience, radiation safety and operational best-practice recommendations have been established to support the safe implementation of Alpha DaRT and provide guidance for source handling, implantation, and post-procedural management [9]. As evidence supporting the clinical safety and efficacy of Alpha DaRT continues to accumulate, attention has increasingly shifted toward factors that may influence successful implementation in routine practice [28]. In addition to demonstrating favorable clinical outcomes, the adoption of a novel treatment modality requires an understanding of the procedural expertise necessary for consistent and reproducible treatment delivery [29]. Evaluation of the learning curve therefore represents an important complementary component of technology assessment, providing insight into how operator experience influences procedural efficiency, performance, and the development of proficiency over time [30]. The present study extends the existing body of evidence on Alpha DaRT by examining the procedural aspects of implementation, thereby complementing prior investigations focused primarily on safety and clinical outcomes. When considered together, assessments of safety, therapeutic effectiveness, and procedural performance provide a more comprehensive understanding of the factors that may influence successful clinical adoption and broader dissemination of Alpha DaRT across diverse clinical settings.

The findings should be interpreted in light of several limitations. The analysis reflects the experience of a single surgeon at a single institution, limiting generalizability. The relatively small sample size restricts statistical power and may obscure subtle associations between lesion characteristics and procedural complexity. In addition, case selection during the early implementation period may not fully represent the spectrum of lesions encountered in routine clinical practice. Variations in tumor size, anatomy, location, and treatment complexity can influence procedure duration and therefore affect learning-curve estimates. Future studies including larger and more heterogeneous patient populations will be important for clarifying the contribution of lesion-specific factors to procedural performance. Additionally, temporal improvements in team coordination or institutional workflow may have contributed to observed efficiency gains. Finally, although this study focused on cutaneous malignancies, Alpha DaRT has broader current and potential oncologic applications. Therefore, the learning curve described here should be interpreted as specific to the cutaneous setting and may not fully reflect the experience associated with other tumor sites or indications.

Nevertheless, these results suggest that Alpha DaRT implantation proficiency, as measured by efficiency, develops relatively rapidly during early clinical use. Future studies involving multiple operators and centers, incorporating additional performance metrics such as implantation accuracy and dosimetric consistency, will be essential to define standardized training requirements and competency benchmarks.

## 5. Conclusions

Alpha DaRT implantation for cutaneous malignancies demonstrates a measurable learning curve, with the greatest gains in procedural efficiency occurring within the first five cases in this single-surgeon experience. While these findings suggest that procedural familiarity develops quickly, the identified five-case proficiency threshold should be considered exploratory and requires confirmation in larger, multi-operator studies before informing training standards. These results contribute to the growing body of evidence supporting the clinical implementation of Alpha DaRT by providing an initial assessment of the procedural experience required to achieve efficient treatment delivery. Characterizing operator learning and procedural performance represents an important complement to existing safety and efficacy data, helping to inform future training strategies, competency benchmarks, and broader adoption of the technology. As additional multicenter and confirmatory validation studies continue to accumulate, they will help clarify the generalizability of these findings and support the safe and consistent integration of Alpha DaRT into routine oncologic practice.

## Figures and Tables

**Table 1:** Descriptive statistics of baseline patient characteristics.

Patient Characteristics	N = 24
Number of Patients	24
Number of Lesions Treated	26
Age (years)—median (range)	71 (37–94)
Gender—N (%)	
Male	15 (62.5%)
Female	9 (37.5%)

**Table 2:** Descriptive statistics of baseline lesion characteristics.

Lesion Characteristics	N = 26
Lesion Status—N (%)	
Newly diagnosed	10 (38%)
Recurrent/persistent	16 (62%)
Lesion Type—N (%)	
BCC	17 (65%)
SCC	9 (35%)
Lesion Locations—N (%)	
Ear	3 (12%)
Forehead	5 (19%)
Lip	3 (12%)
Nose	6 (23%)
Scalp	5 (19%)
Other	4 (15%)
Longest Lesion Diameter (cm)—mean (range)	1.77 (1.0–4.17)
Gross Tumor Volume (cm^3^)—mean (range)	1.14 (0.06–7.31)

**Table 3:** Descriptive statistics of procedure characteristics.

Procedure Characteristics	*N* = 26
Sources Inserted—median (range)	29.5 (12–91)
Applicators Used—median (range)	11.5 (6–47)
Duration/Applicator in sec. Procedures 1–5—mean, SD [95% CI]	221, 58 [148.9–293.3]
Duration/Applicator in sec. Procedures 6–26—mean, SD [95% CI]	153, 29 [139.9–166.7]

## Data Availability

The data supporting the findings of this study may be obtained upon request from the corresponding author.

## Supplementary Materials

The following supporting information can be downloaded at: https://www.mdpi.com/article/10.3390/cancers18213425/s1, Table S1: Raw dataset.

## Abbreviations

The following abbreviations are used in this manuscript:

DaRT	Diffusing Alpha-emitters Radiation Therapy
BCC	Basal-Cell Carcinoma
CI	Confidence Interval
SCC	Squamous-Cell Carcinoma
SD	Standard Deviation
LET	Linear Energy Transfer
